# Progress of Home-Based Food Allergy Treatment during the Coronavirus Disease Pandemic in Japan: A Cross-Sectional Multicenter Survey

**DOI:** 10.3390/children8100919

**Published:** 2021-10-15

**Authors:** Akihiro Maeta, Yuri Takaoka, Atsuko Nakano, Yukiko Hiraguchi, Masaaki Hamada, Yutaka Takemura, Tomoko Kawakami, Ikuo Okafuji, Makoto Kameda, Kyoko Takahashi

**Affiliations:** 1 Department of Food Science and Nutrition, School of Food Science and Nutrition, Mukogawa Women’s University, 6-46, Ikebiraki-cho, Nishinomiya 663-8558, Japan; maeta_a@mukogawa-u.ac.jp; 2 Department of Pediatrics, Osaka Prefectural Hospital Organization Osaka Habikino Medical Center, 3-7-1, Habikino 583-8588, Japan; zvb11075@nifty.com (Y.T.); kamedam@ra.opho.jp (M.K.); 3 Department of Pediatrics, Kokuho Chuo Hospital, 404-1, Miyako, Shiki 636-0302, Japan; atsuko.nah@hotmail.com; 4 Department of Pediatrics, Osaka Saiseikai Nakatsu Hospital, 2-10-39, Shibata, Osaka 530-0012, Japan; hiraguchiy7240@gmail.com; 5 Department of Pediatrics, Yao Municipal Hospital, 1-3-1, Ryuge-cho, Yao 581-0069, Japan; hssd1895@yahoo.co.jp; 6 Department of Pediatrics, Kindai University Hospital, 377-2, Onohigashi, Osakasayama 577-8502, Japan; ytktkmr@med.kindai.ac.jp; 7 Department of Pediatrics, Sumitomo Hospital, 5-3-20, Nakanoshima, Osaka 530-0005, Japan; kawakami-tomoko@sumitomo-hp.or.jp; 8 Department of Pediatrics, Kobe City Hospital Organization Kobe City Medical Center General Hospital, 2-1-1, Minatoshima-cho, Kobe 650-0047, Japan; ikuoka@gmail.com

**Keywords:** SARS-CoV-2, food allergy treatment, oral immunotherapy, COVID-19, cross-sectional studies

## Abstract

The coronavirus disease 2019 (COVID-19) pandemic’s impact on food allergy treatment such as home-based oral immunotherapy (OIT) is not known. This cross-sectional, questionnaire-based anonymized survey screened 2500 parents of children with allergic diseases and was conducted in the pediatric outpatient clinics of 24 hospitals. Basic clinical data of the children were collected along with the degree of allergy control, parental anxiety about emergency visits, and the risk of COVID-19 in the first state of emergency. A total of 2439 (97.6%) questionnaires were collected, and 1315 parents who were instructed to initiate home-based OIT for their children were enrolled (OIT group). Subjective OIT progress compared to before the COVID-19 pandemic was ascertained as “Full”, “Middle”, “Low”, “Little”, and “Stop” in 264 (20.1%), 408 (31.0%), 384 (29.2%), 203 (15.4%), and 56 (4.3%) participants, respectively. Anxiety about emergency visits and the risk of COVID-19 were negatively associated with the subjective OIT progress. In Japan, approximately half of the children continued smoothly the home-based OIT during the COVID-19 pandemic. Parents with high levels of anxiety about the disruption of the medical care system due to COVID-19 and the risk of COVID-19 did not experience a smooth continuation of home-based OIT.

## 1. Introduction

Since November 2019, coronavirus disease 2019 (COVID-19) has had a profound impact on routine human life [1]. On 11 March 2020, the World Health Organization declared COVID-19 a pandemic. Several countries, such as the United States and France, implemented lockdowns with penalties to contain the COVID-19 spread. In Japan, educational institutions suspended operations on 2 March 2020 [2]. In seven Japanese cities (Tokyo, Kanagawa, Chiba, Saitama, Osaka, Hyogo, and Fukuoka), the first state of emergency without penalties was declared on 7 April 2020 [3]; this was extended nationwide on 16 April 2020. From 2 March 2020 to 16 April 2020, the numbers of daily and cumulative cases rose exponentially, to 4478 and 9362 cases, respectively (Supplementary Figure S1). With the goal of reducing contact between people by 70–80%, the first state of emergency was implemented to prevent people from going out unnecessarily, to promote teleworking, and to request that business owners suspend operations [3]. As a result of the state of emergency, the number of people around Osaka Station was reduced by approximately 80% and 90% on weekdays and holidays, respectively, compared to the numbers before the spread of COVID-19 [4]. Moreover, the leave rates for kindergartens, elementary schools, junior high schools, and high schools were 74%, 95%, 95%, and 97%, respectively [5]. This state of emergency lasted approximately 1–2 months, and many people, including children, stayed at home throughout.

Oral immunotherapy (OIT) has been widely used as a novel treatment for food allergy (FA) [6]. In the Japanese Guidelines for Food Allergy 2020 [6], OIT is defined as a treatment method for cases where the early acquisition of tolerance during the natural course cannot be anticipated. After a symptom induction threshold has been determined during an earlier oral food challenge, causative foods are taken under a physician’s instruction, aiming to acquire the conditions of increased threshold or desensitization [6]. In Japan, the number of patients receiving OIT increased approximately sixfold from 2012 to 2015 [6]. OITs are continued at home [6,7,8], despite the ingestion of food antigens posing a risk of adverse reaction. Therefore, in home-based OIT, it is essential to prescribe therapeutic drugs, including adrenaline and antihistaminic medicines and to expand safety measures such as 24-h emergency consultation and cooperation with local emergency medical institutions. The continuation of OIT greatly affects not only the patient but also the parents’ willingness to treat their children. Anxiety about dealing with allergic reactions and about a 24-h emergency consultation system is related to psychological burdens for parents [9]. In an anonymous and voluntary online cross-sectional survey in Australia, it was reported that COVID-19 pandemic made it difficult to access new food experiences and FA-related health services [10]. Thus, it was considered that the spread of COVID-19 is one of factors preventing the smooth continuation of OIT given the increase in psychological burden on parents.

The relationship between the COVID-19 pandemic and FA treatments has been explored previously [10,11,12,13,14,15,16,17,18], although these reports were review articles (not surveys) [11,12,13,14,15,16,17] or small-scale surveys that included patients and their parents [10,18,19]. However, no large-scale study comprising more than 1000 subjects has evaluated the relationship between the COVID-19 pandemic and FA treatment. We hypothesized that the food antigen exposure process would be disrupted by the COVID-19 pandemic. Therefore, we aimed to explore the subjective progression of home-based OIT by the COVID-19 pandemic on the perspective of the parent’s psychological state.

## 2. Materials and Methods

### 2.1. Facilities, Subjects, and Ethical Considerations

This cross-sectional, anonymized survey was conducted in the pediatric outpatient clinics of 24 hospitals in Osaka, Hyogo, and Nara from October 2020 to April 2021 (Supplementary Table S1) with the approval of each hospital and the Research Ethics Committee of Mukogawa Women’s University (approval number: 20–65). The parents/guardians of the children with allergic diseases were fully informed about the survey verbally and in writing. The submission of a questionnaire was considered to be consent in the survey. The participants were 2500 parents continually going to the pediatric outpatient clinics of 24 hospitals to treat their children (aged 0–15 years) with allergic diseases. In this survey, we defined OIT as a FA treatment that involves ingesting the causative food at home to increase the threshold of food allergen.

### 2.2. Questionnaire

The original contents of the questionnaire were designed by pediatricians, and survey responses were anonymized (Supplementary Figures S2 and S3). The questionnaire assessed the following: two questions about FA treatment for children (FQ1 and FQ2); four questions about the parents’ anxiety about visiting the hospital, ambulatory care, and the risk of severe acute respiratory syndrome coronavirus 2 (SARS-CoV-2) infection in the first state of emergency (Q1–Q4); the Japanese-translated State-Trait Anxiety Inventory (STAI) [20]; and basic clinical and epidemiological information of the children with allergic diseases. 

The influence on FA treatments of the COVID-19 pandemic (FQ2) was determined by the subjective evaluation of parents on the progress of home-based OIT during the spread. The FQ2 asked parents who had children carrying out OIT at home for the comparison to before the spread of COVID-19. The subjective progress of home-based OIT was rated with five options: “Full progress as planned” (Full), “Middle progress not as planned” (Middle), “Low progress not as planned” (Low), “Little progress” (Little), and “Stop of the treatment” (Stop) were included. Moreover, we investigated the reasons for progress and non-progress using a free-text descriptive formula. 

For answers to questions about the parents’ anxiety (Q1–Q4) regarding emergency visits, ambulatory care, and SARS-CoV-2 infection, the options were: “None”, “Not really”, “Some”, “A lot”, and “Quite a lot”. 

Anxietas (anxiety as a personality trait) was measured using the STAI [20].

### 2.3. Inclusion/Exclusion Criteria

**Figure 1:**
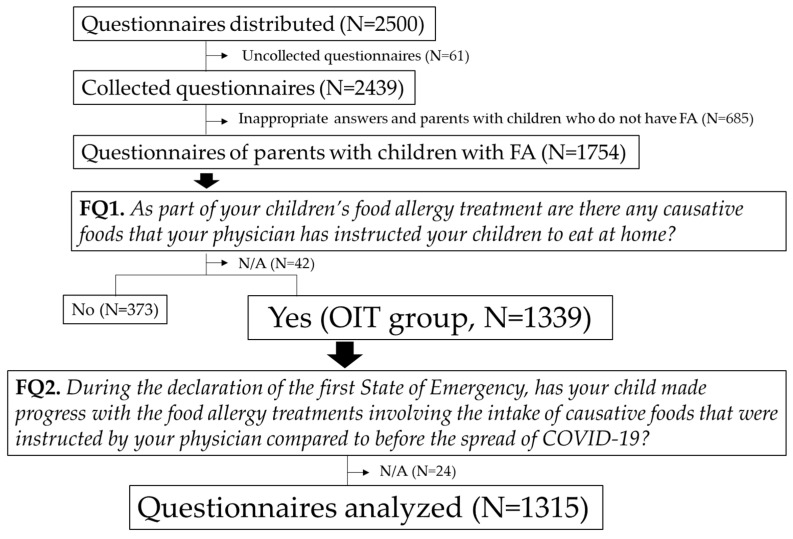
Flowchart of the participant selection process employed in this study.

Inclusion criteria of the OIT group is a “Yes” answer to FQ1. The flow chart of the selection of analyzed questionnaires is shown in Figure 1. Exclusion criteria for the final analysis was parents with children who do not have FA, parents with children who do not carry out home-based OIT (FQ1), and parents who did not answer FQ1 and FQ2.

### 2.4. Statistical analyses

Children’s ages and the STAI of the parents are presented as medians and interquartile ranges. All other data are presented as numbers and percentages.

In the stratified analysis of the parents’ anxiety (Q1–Q4), we included “None” and “Not really” in the low-anxiety (small) group; “Some” in the medium-anxiety (medium) group; and “A lot” and “Quite a lot” in the high-anxiety (large) group.

In males and females, an anxiety score of more than 44 or 45 points, respectively, was indicative of high anxietas [20]. 

Statistical analyses were conducted using the chi-square test. The family-wise error rate was corrected using Bonferroni’s method. Differences were considered significant at *p* < 0.05. All analyses were conducted using GraphPad Prism (version 5.0; La Jolla, CA, USA).

## 3. Results

### 3.1. Participant Information and Subjective Progress of Home-Based OIT

A flowchart of the participant selection process is shown in Figure 1. 

A total of 2439 questionnaires were collected (response rate: 97.6%). Among these, we excluded the questionnaires of parents who did not have children with FA, which left 1754 completed questionnaires from parents of children with FA. Of these, 1339 questionnaires were from parents who were instructed to conduct home-based OIT for their children (OIT group). The final analysis included 1315 questionnaires, after excluding 24 questionnaires that did not include answers to the question about the progress of causative food intake (FQ2). The baseline information of the children with FA and their parents is shown in Table 1.

**Figure 2:**
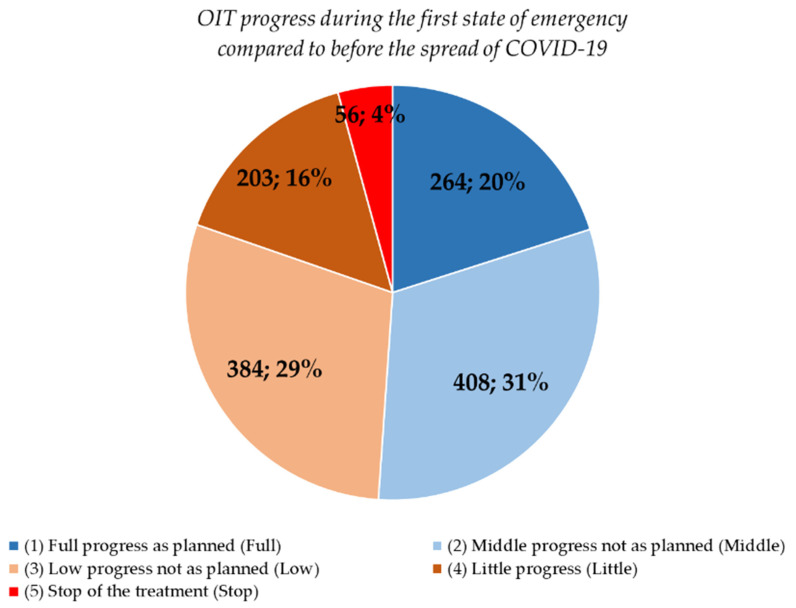
Progress of home-based oral immunotherapy (OIT) in the first state of emergency. Data are shown as the number of respondents and percentage (parents).

Compared to before the COVID-19 pandemic, the question about the subjective progress of home-based OIT (FQ2) elicited the following responses: “Full” in 264 (20.1%) respondents, “Middle” in 408 (31.0%), “Low” in 384 (29.2%), “Little” in 203 (15.4%), and “Stop” in 56 (4.3%) (Figure 2). The analysis of the free-text descriptive responses of the reasons for “Full” or “Middle” showed that the frequency of the appearance of “at home”, “time”, and “increased” was high, but the reasons for “Low”, “Little”, or “Stop” showed that the frequency of “emergency”, “consultation”, “scary”, and “symptom” was high (data not shown for both).

### 3.2. Relationship between the Participant’s Information and the Subjective Progress of Home-Based OIT

Stratified analyses showed no associations between the subjective progress of home-based OIT and age of the child, the prevalence of allergic diseases other than FA, the history of anaphylaxis, and the location of the outpatient hospitals; however, there was a significant association between the prescription of adrenaline and the progress of home-based treatment (Table 2). The percentage of respondents who answered “Full”, “Little”, and “Stop” for the OIT progress question was higher in those who had a prescription for adrenaline than in those who did not have a prescription (Table 2).

### 3.3. Relationship between Parents’ Anxiety about Emergency Visits, Ambulatory Care, and SARS-CoV-2 Infections and the Subjective Progress of Home-Based OIT

For all questions (Table 3), parents in the low- and medium-anxiety groups had more “Full” and “Middle” progress compared with those in the high-anxiety group. In Q1 and Q3 (questions about emergency or outpatient treatment), the proportion of respondents who answered “Low”, “Little”, and “Stop” in the high-anxiety group was higher than those in the low- and medium-anxiety groups (Table 3). In Q2 and Q4 (questions related to anxieties about COVID-19), the proportion of respondents who answered “Low”, “Little”, and “Stop” was higher in the high-anxiety group than those in the low- and medium-anxiety groups (Table 3). Moreover, in Q2 and Q4, the most common answer in the low-anxiety group was “Middle”, while “Low” was the most common answer in the medium-anxiety group (Table 3).

### 3.4. Relationship between Parents’ Anxietas (Anxiety as a Personality Trait) and the Subjective Progress of Home-Based OIT

**Figure 3:**
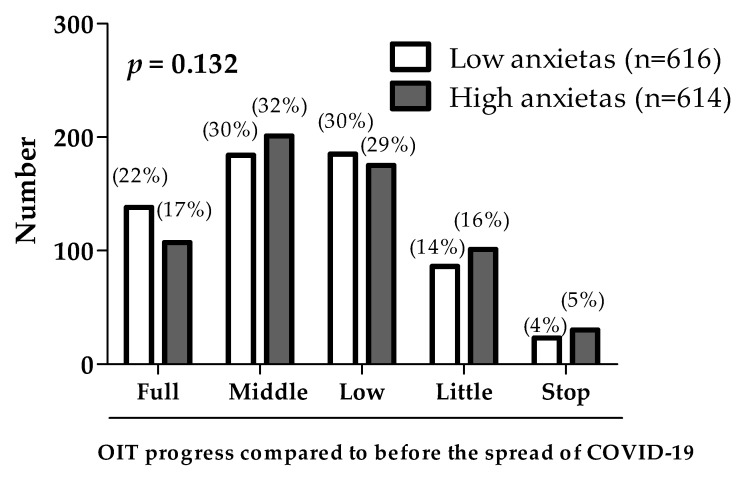
Relationship between anxietas (anxiety as a personality trait) of parents and the progress of home-based oral immunotherapy (OIT). Data are shown as the number of respondents and percentage (parents).

The STAI had 1230 (93.5%) and 85 (6.5%) complete and incomplete answers, respectively. The proportion of respondents with high anxietas comprised approximately half of the study population (49.9%, 614/1230). There was no association between the anxietas groups of parents and the subjective progression of home-based OIT (Figure 3).

## 4. Discussion

The COVID-19 pandemic has profoundly impacted daily life worldwide [1]. In Japan, the state of emergency without penalties was first declared in April 2020 [3]. However, with the COVID-19 pandemic disrupting medical practice and the restriction of outings under the state of emergency, it was unclear how parents managed home-based OIT for their children. Therefore, we aimed to explore the subjective progression of home-based OIT by the COVID-19 pandemic on the perspective of the parent’s psychological state.

The home-based OIT requires ingestion of the causative food antigen at home. However, the ingestion of food allergenic products for OIT poses the risk of an adverse reaction [6]. Ozturk et al. reported that only 21% of the respondents were continuing subcutaneous immunotherapy as usual during the COVID-19 pandemic [18]. Krishna et al. reported that there was a highly significant decrease in subcutaneous immunotherapy up dosing and maintenance during the pandemic [19]. We predicted that many parents would temporarily suspend the home-based OIT for children with FA to avoid the risk of emergency visits and ambulatory care for adverse reactions. However, the rate of discontinuation of OIT (“Stop”) was less than 5%, even in the first state of emergency. Moreover, approximately half of the patients were able to make complete or partial progress with home-based OIT compared to that before the COVID-19 pandemic. From the analysis of the free-text descriptions, the reason for the smooth continuation of home-based OIT was most likely due to the increased time spent at home. After children with FA ingest the food antigen, parents need to monitor the condition of their children due to the likelihood of an induced allergic reaction [6]. Papadopoulos et al. reported that outcomes in pediatric asthma may even have improved by increased adherence and/or reduced exposure during the COVID-19 pandemic [21]. Therefore, we considered the possibility that the increased time spent at home due to the state of emergency contributed to the increased observation time after the children with FA had ingested the food antigen. This was the reason for the smooth continuation of OIT.

However, approximately half of the patients (“Low”, “Little”, and “Stop”) were unable to continue smoothly the home-based OIT or had to discontinue it. We explored factors that were related to the OIT progress and found a significant association between the prescription of adrenaline and the ability to progress in home-based OIT. The parents of children with FA who were prescribed adrenaline tended to have more difficulties continuing the home-based OIT. Adrenaline is prescribed to children with severe FA. Therefore, we considered it likely that the parents decided not to proceed with OIT to avoid the risk of serious allergic reactions at home. Chen et al. and Tagami et al. reported that the number of children with an immediate allergic reaction to foods decreased during COVID-19 pandemic [10,22]. From the stratified analysis and analysis of free-text descriptions, parents who experienced strong anxiety against ambulatory visits, emergency care, and risk of SARS-CoV-2 infection tended to be less able to continue smoothly the home-based OIT. However, there was no association between a parent’s anxiety as a personality trait and the OIT progress. Parents of children with severe FA, who are at a high risk of requiring emergency care after consuming the causative antigen, may have been more sensitive to the disruption of the medical care system. 

Furthermore, we found an interesting association between the prescription of adrenaline and the subjective progress of home-based OIT. A higher proportion of the group of parents of children who were prescribed adrenaline answered “Full” to the question about the progress of home-based OIT than those of children who were not prescribed adrenaline. Injectable adrenaline is an essential emergency treatment that parents can administer if an anaphylactic shock occurs following the intake of causative food antigens [6]. Thus, we inferred that the prescription of adrenaline gave parents a sense of relief. Parents with low anxiety about the confusing medical care systems and risks of SARS-CoV-2 infection tended to experience the smooth continuation of home-based OIT. For OIT progression, it is important to dispel excessive anxiety about the COVID-19 pandemic in the parents and teach them to administer emergency treatments for severe FA-treatment-related adverse symptoms. Therefore, to ensure the smooth continuation of home-based OIT during a pandemic, it may be important for physicians to address parental anxieties regarding OIT by providing accurate information about COVID-19, the availability of hospital services, and by prescribing essential medicines.

This study has some limitations. First, this study has selection bias because the participants of this survey were the parents of children who continued to seek consultation for the treatment of allergic diseases. Parents who did not continue to seek consultation for their children following the onset of the COVID-19 pandemic were not included. Thus, the number of discontinuations of OIT may be higher than the results of this survey. Second, this survey was conducted only in Japan. From March to May 2020, the cumulative number of SARS-CoV-2 infections was lower in Japan than in other developed countries, such as the United States and European countries. Therefore, it is possible that the impact of the COVID-19 pandemic was smaller in Japan than in other developed countries. Third, the possibility of recall bias exists as the parents’ memories may have affected their answers. Fourth, we did not clearly define the progression of OIT because OIT methods with food antigen ingestion vary from facility to facility and from patient to patient. However, FQ2 (the subjective progression of home-based OIT) asked the parents who had children carrying out OIT at home for the comparison of conditions before the spread of COVID-19. Therefore, we considered that FQ2 (the subjective progression of home-based OIT) could evaluate relative progress in in the subjectivity of parents compared to before the spread of COVID-19. Fifth, because the survey was anonymous, the patient’s medical records and the completed questionnaire could not be linked. The information provided by parents may not match the child’s correct diagnosis record. However, we considered that most children with FA in this study were diagnosed by an oral food challenge test because the determination of their intake threshold to allergen foods is needed to conduct home-based OIT.

## 5. Conclusions

In Japan, home-based OIT was continued during the COVID-19 pandemic, and half of parents were able to comply with OIT for their children. Parents with high anxiety about the disruption of medical care systems were more likely to discontinue home-based OIT. Furthermore, preventing pandemics is essential for the management of diseases other than infectious diseases.

## Figures and Tables

**Table 1:** Background information of parents and children.

Item	Number (%) or Median (25th–75th Percentile) ^1^
Parents	1315
Relationship	
Father	90 (6.8%)
Mother	1216 (92.5%)
Other or no answer (N/A)	9 (0.7%)
STAI of parents (N = 1230)	
Total score	91 (80–105)
Angor (anxiety as a now state)	46 (40–54)
Anxietas (anxiety as a personality trait)	44 (38–53)
Children with FA	1429
Age, year (N = 1414)	6 (3–9)
Sex	
Boys	942 (65.9%)
Girls	481 (33.7%)
N/A	8 (0.6%)
Prevalence of allergic diseases other than FA (multiple answers allowed)	
Bronchial asthma	279 (19.5%)
Atopic dermatitis	580 (40.6%)
Allergic rhinitis	315 (22.0%)
Allergic conjunctivitis	63 (4.4%)
History of anaphylaxis	247 (17.3%)
Prescription of adrenaline	570 (39.9%)
Oral immunotherapy food (multiple answers allowed)	
Hen’s egg	834 (58.4%)
Cow’s milk	481 (33.7%)
Wheat	218 (15.3%)
Other (such as peanuts, soybean and etc.)	56 (4.0%)

^1^ Data on age of children and State-Trait Anxiety Inventory (STAI) of parents are presented as median and interquartile range. All other data are shown as number and percentage. N/A; No answer.

**Table 2:** Relationship between the subject’s information and the oral immunotherapy (OIT) progress during the first state of emergency.

		OIT Progress Compared to before the Spread of COVID-19					*p*-Value (χ^2^-Test)
		Full	Middle	Low	Little	Stop	
Have at least child with FA over 6 years old							
Yes	N = 693	138 (19.9%)	195 (28.1%)	211 (30.4%)	115 (16.6%)	34 (4.9%)	0.094
No	N = 609	123 (20.2%)	210 (34.5%)	169 (27.8%)	86 (14.1%)	21 (3.4%)	
N/A	N = 13						
Prevalence of allergic diseases other than FA							
Only FA	N = 600	132 (22.0%)	195 (32.5%)	167 (27.8%)	85 (14.2%)	21 (3.5%)	0.188
Other diseases	N = 715	132 (18.5%)	213 (29.8%)	217 (30.3%)	118 (16.5%)	35 (4.9%)	
History of anaphylaxis							
Yes	N = 233	45 (19.3%)	61 (26.2%)	70 (30.0%)	40 (17.2%)	17 (7.3%)	0.062
No	N = 1082	219 (20.2%)	347 (32.1%)	314 (29.0%)	163 (15.1%)	39 (3.6%)	
Prescription of adrenaline							
Yes	N = 549	122 (22.2%)	146 (26.6%)	153 (27.9%)	98 (17.9%)	30 (5.5%)	0.004
No	N = 766	142 (18.5%)	262 (34.2%)	231 (30.2%)	105 (13.7%)	26 (3.4%)	
Location of outpatient hospital							
Osaka	N = 974	207 (21.3%)	297 (30.5%)	283 (29.1%)	145 (14.9%)	42 (4.3%)	0.734
Hyogo	N = 132	24 (18.2%)	41 (31.1%)	42 (31.8%)	21 (15.9%)	4 (3.0%)	
Nara	N = 209	33 (15.8%)	70 (33.5%)	59 (28.2%)	37 (17.7%)	10 (4.8%)	

Data (N = 1315) are presented as the number and the percentage of respondents (parents) in each group. N/A; No answer.

**Table 3:** Relationship between the subject’s information and the oral immunotherapy (OIT) progress during the first state of emergency.

		OIT Progress Compared to before the Spread of COVID-19					*p*-Value (χ^2^-Test)	
		Full	Middle	Low	Little	Stop		
Q1. Did you experience any anxiety as to whether you would be able to have normal consultations at the hospital (outpatient or emergency)?								
Low anxiety “None” or “Not really”	N = 153	50 (32.7%)	46 (30.1%)	36 (23.5%)	16 (10.5%)	5 (3.3%)	Small vs. Medium	0.116
Medium anxiety “Some”	N = 450	98 (21.8%)	152 (33.8%)	127 (28.2%)	66 (14.7%)	7 (1.6%)	Small vs. Large	<0.001
High anxiety “A lot” or “Quite a lot”	N = 709	116 (16.4%)	209 (29.5%)	220 (31.0%)	120 (16.9%)	44 (6.2%)	Medium vs. Large	<0.001
N/A	N = 3							
Q2. Did you worry that by going to the hospital, your family could become infected with COVID-19?								
Low anxiety “None” or “Not really”	N = 153	46 (30.1%)	57 (37.3%)	28 (18.3%)	20 (13.1%)	2 (1.3%)	Small vs. Medium	0.018
Medium anxiety “Some”	N = 422	90 (21.3%)	131 (31.0%)	142 (33.6%)	50 (11.8%)	9 (2.1%)	Small vs. Large	<0.001
High anxiety “A lot” or “Quite a lot”	N = 738	128 (17.3%)	219 (29.7%)	214 (29.0%)	132 (17.9%)	45 (6.1%)	Medium vs. Large	0.002
N/A	N = 2							
Q3. Have you ever worried about the opinions of others and thought about postponing or cancelling a scheduled outpatient appointment/test/inpatient treatment?								
Low anxiety “None” or “Not really”	N = 597	148 (24.8%)	200 (33.5%)	149 (25.0%)	81 (13.6%)	19 (3.2%)	Small vs. Medium	0.461
Medium anxiety “Some”	N = 349	63 (18.1%)	124 (35.5%)	104 (29.8%)	47 (13.5%)	11 (3.2%)	Small vs. Large	<0.001
High anxiety “A lot” or “Quite a lot”	N = 367	53 (14.4%)	83 (22.6%)	131 (35.7%)	74 (20.2%)	26 (7.1%)	Medium vs. Large	<0.001
N/A	N = 2							
Q4. Have you ever experienced anxiety that, due to allergies, a COVID-19 infection could become worse?								
Low anxiety “None” or “Not really”	N = 647	145 (22.4%)	229 (35.4%)	172 (26.6%)	86 (13.3%)	15 (2.3%)	Small vs. Medium	0.036
Medium anxiety “Some”	N = 287	48 (16.7%)	82 (28.6%)	102 (35.5%)	47 (16.4%)	8 (2.8%)	Small vs. Large	<0.001
High anxiety “A lot” or “Quite a lot”	N = 379	71 (18.7%)	96 (25.3%)	110 (29.0%)	69 (18.2%)	33 (8.7%)	Medium vs. Large	0.037
N/A	N = 2							

Data (N = 1315) are presented as the number and the percentage of respondents (parents) in each group. N/A; No answer.

## Supplementary Materials

The following are available online at https://www.mdpi.com/article/10.3390/children8100919/s1, Table S1: Surveyed Hospitals; Figure S1: The number of individuals infected with the severe acute respiratory syndrome coronavirus 2 in Japan from 24 February to 1 June 2020; Figure S2: Questionnaire assessing progress of food allergy treatment (FQ1 and 2) and parents’ anxiety about visiting hospital, ambulatory care, and SARS-CoV-2 infection (Q1–Q4) in the first state of emergency; Figure S3: Questionnaire assessing the child’s epidemiological and clinical information.

## Appendix A. List of Primary Investigators and Co-Investigators

Primary Investigators
  Akagawa Shohei MD, PhD ^1^, Anzai Kaori, MD ^2^, Bandou Kenji, MD ^3^, Ikeda Akiko, MD ^4^, Doi Masaaki, MD, PhD ^5^, Enomoto Masahiro, MD, PhD ^6^, Fujikawa Shiori, MD ^7^, Nagai Megumi MD, PhD ^8^, Nishiyama Atsuko, MD ^9^, Otsuka Keita, MD ^10^, Shimizu Satoko, MD ^11,12^, Sugimoto Yukiko, MD ^13^, Sumimoto Shinichi, MD, PhD ^2^, Tanaka Yukiko, MD ^14^, Tanaka Yuko, MD ^15^, Tanaka Yuya, MD ^16^, Wakahara Ryohei, MD, PhD ^17^, Yamasaki Koji, MD, PhD ^18^
Co-Investigators
  Arima Tomoyuki, MD ^8^, Imaide Aya, MD ^6^, Fukasawa Yohei, MD ^21^, Hashimoto Naoki, MD ^19^, Masumi Hiroki MD ^8^, Matsutani Eri, MD ^3^, Kim Jong Soo, MD ^20^, Nakai Yoko, MD ^1^, Nakamichi Erina, MD ^2^, Natsuki Momo MD ^16^, Onaka Masayuki, MD ^9^, Shingaki Tomoya, MD ^20^, Sunaga Ayana, MD ^3^, Tsurinaga Yuki, MD ^21^, Yamada Saki, MD ^20^, Yamagishi Mitsuru, MD ^1^

^1^ Kansai Medical University Hospital
^2^ Japanese Osaka Red Cross Hospital
^3^ Izumi City General Hospital
^4^ Yamatotakada Municipal Hospital
^5^ Higashiosaka City Medical Center
^6^ Takatsuki General Hospital
^7^ Abeno Medical Clinic
^8^ Kindai University Hospital
^9^ Nara Prefecture General Medical Center
^10^ Nara City Hospital
^11^ Matsushita Memorial Hospital
^12^ Shimizu Family Clinic
^13^ Hoshigaoka Medical Center
^14^ Kobe City Medical Center West Hospital
^15^ Osaka Police Hospital
^16^ Hyogo Prefectural Kobe Children’s Hospital
^17^ PL Hospital
^18^ Kaizuka City Hospital
^19^ Kokuho Chuo Hospital
^20^ Osaka Saiseikai Nakatsu Hospital
^21^ Osaka Habikino Medical Center
