# Supplementary material for: Progress of Home-Based Food Allergy Treatment during the Coronavirus Disease Pandemic in Japan: A Cross-Sectional Multicenter Survey
Source: Children (Basel). 2021 Oct 15;8(10):919. doi: 10.3390/children8100919 (PMC8535074; doi:10.3390/children8100919)
Supplement: Supplementary file 1 [file children-08-00919-s001.zip › Supple_Fig_1-3.pdf]

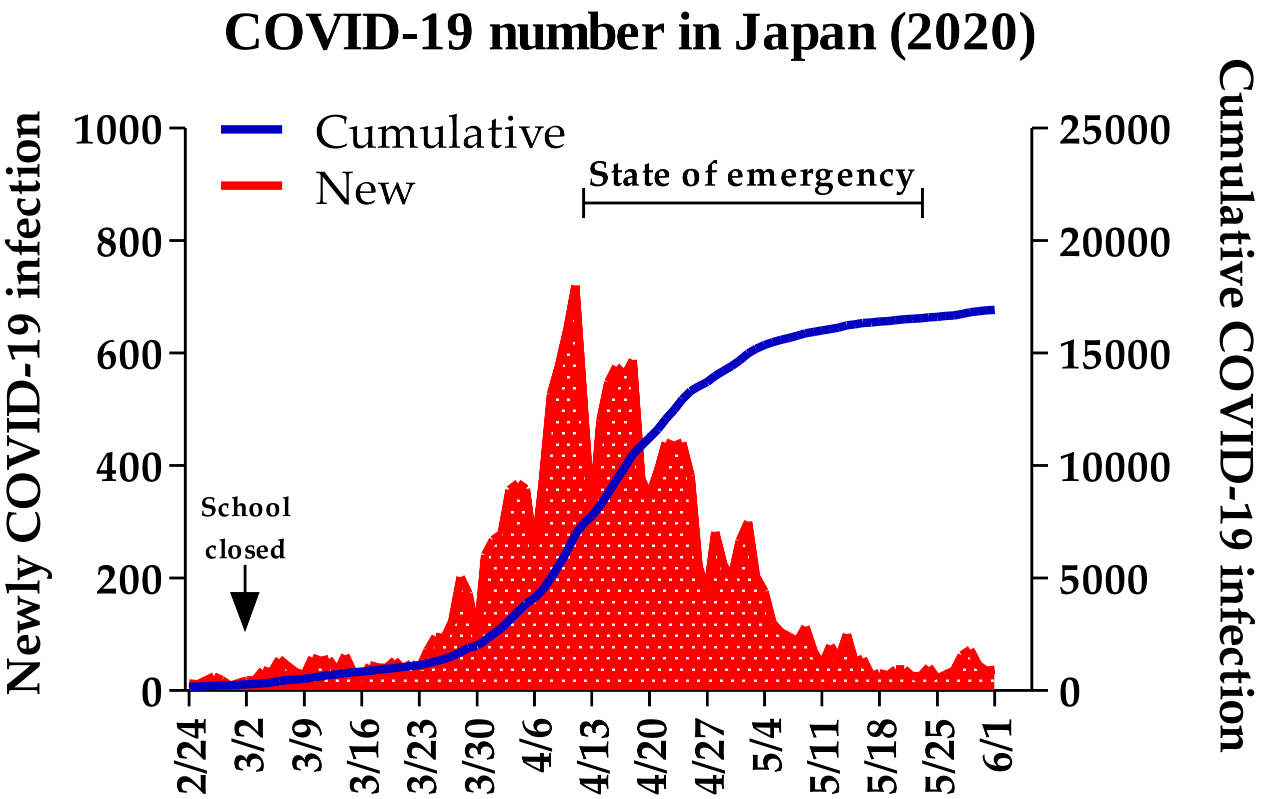

Supplementary Figure S1. The number of individuals infected with the severe acute respiratory syndrome coronavirus 2 in Japan from February 24 to June 1, 2020.

The data used were compiled by the Japan Broadcasting Corporation.

FQ1. As part of your children's food allergy treatment are there any causative foods that your physician has instructed your children to eat at home?	(1) Yes ⇒ Go to FQ2<br>(2) No ⇒ Go to Q1

FQ2. During the declaration of the first state of emergency, has your child made progress with their food allergy treatment involving the intake of causative foods that were instructed by your physician compared to before the spread of COVID-19?	(1) Full progress as planned ⇒ Go to FQ2-1<br>(2) Middle progress not as planned ⇒ Go to FQ2-1<br>(3) Low progress not as planned ⇒ Go to FQ2-2<br>(4) Little progress ⇒ Go to FQ2-2<br>(5) Stop of the treatment ⇒ Go to FQ2-2
FQ2-1. Please tell us why your child has made progress.	
FQ2-2. Please tell us why your child has not made progress, or why they have ceased treatment.	

Q1. During the declaration of the first state of emergency, did you experience any anxiety as to whether you would be able to have normal consultations at the hospital (outpatient or emergency)?	(1) None<br>(2) Not really<br>(3) Some<br>(4) A lot<br>(5) Quite a lot

Q2. During the declaration of the first state of emergency, did you worry that by going to the hospital, your family could become infected with COVID-19?	(1) None<br>(2) Not really<br>(3) Some<br>(4) A lot<br>(5) Quite a lot

Q3. During the declaration of the first state of emergency, have you ever worried about the opinions of others and thought about postponing or cancelling a scheduled outpatient appointment/test/inpatient treatment?	(1) None<br>(2) Not really<br>(3) Some<br>(4) A lot<br>(5) Quite a lot

Q4. During the declaration of the first state of emergency, have you ever experienced anxiety that due to allergies, a COVID-19 infection could become worse?	(1) None<br>(2) Not really<br>(3) Some<br>(4) A lot<br>(5) Quite a lot

Supplementary Figure S2. Questionnaire assessing progress of food allergy treatment (FQ1 and 2) and parents' anxiety about visiting hospital, ambulatory care, and SARS-CoV-2 infection (Q1-Q4) in the first state of emergency

C1. Age of child who have sustained consultation due to allergic diseases
(0) (1) (2) (3) (4) (5) (6) (7) (8) (9) (10) (11) (12) (13) (14) (15) year
(0) (1) (2) (3) (4) (5) (6) (7) (8) (9) (10) (11) months

C2. Sex of child who have sustained consultation due to allergic diseases	(1) Boy<br>(2) Girl

C3. Which allergic disease has your child?<br>(Multiple answers allowed)	(A) Food allergy<br>(B) Anaphylaxis<br>(C) Bronchial asthma<br>(D) Atopic dermatitis<br>(E) Allergic rhinitis<br>(F) Allergic conjunctivitis

C4. Which medicine does your child normally use?<br>(Multiple answers allowed)	(A) Adrenaline self-injection<br>(B) Inhaled steroids<br>(C) Leukotriene antagonist<br>(D) Biopharmacy<br>(E) Topical steroids<br>(F) Moisturizer<br>(G) Antihistamine<br>(H) Nasal drops for allergic rhinitis<br>(I) Cedar sublingual immunotherapy<br>(J) Tick sublingual immunotherapy<br>(K) Eye drops for the treatment of allergic conjunctivitis<br>(L) Others（ ）

C5. Which immunotherapy has your child carried out? (Multiple answers allowed)	(A) Hen's egg<br>(B) Cow's milk<br>(C) Wheat<br>(D) Cedar<br>(E) Tick<br>(F) Others（ ）

Supplementary Figure S3. Questionnaire assessing the child's epidemiological and clinical information.
