# Supplementary material for: Progress of Home-Based Food Allergy Treatment during the Coronavirus Disease Pandemic in Japan: A Cross-Sectional Multicenter Survey
Source: Children (Basel). 2021 Oct 15;8(10):919. doi: 10.3390/children8100919 (PMC8535074; doi:10.3390/children8100919)
Supplement: Supplementary file 1 [file children-08-00919-s001.zip › Supple_Table_1.pdf]

Supplementary Table S1. Surveyed Hospitals

No	Hospital lists	Location	Analyzed Number (%)
1	Hyogo Prefectural Kobe Children's Hospital	Hyogo	70 (5.3%)
2	Kansai Medical University Hospital	Osaka	71 (5.4%)
3	Kindai University Hospital	Osaka	46 (3.5%)
4	Higashiosaka City Medical Center	Osaka	37 (2.8%)
5	Izumi City General Hospital	Osaka	14 (1.1%)
6	Sumitomo Hospital	Osaka	57 (4.3%)
7	Japanese Osaka Red Cross Hospital	Osaka	21 (1.6%)
8	Matsushita Memorial Hospital	Osaka	1 (0.1%)
9	Yamatotakada Municipal Hospital	Nara	34 (2.6%)
10	Takatsuki General Hospital	Osaka	74 (5.6%)
11	Kobe City Medical Center General Hospital	Hyogo	51 (3.9%)
12	Osaka Saiseikai Nakatsu Hospital	Osaka	155 (11.8%)
13	Yao Municipal Hospital	Osaka	78 (5.9%
14	Kobe City Medical Center West Hospital	Hyogo	11 (0.8%)
15	PL Hospital	Osaka	6 (0.5%)
16	Kokuho Chuo Hospital	Nara	122 (9.3%)
17	Nara City Hospital	Nara	38 (2.9%)
18	Hoshigaoka Medical Center	Osaka	5 (0.4%)
19	Abeno Medical Clinic	Osaka	13 (1.0%)
20	Kaizuka City Hospital	Osaka	60 (4.6%)
21	Osaka Police Hospital	Osaka	9 (0.7%)
22	Nara Prefecture General Medical Center	Nara	15 (1.1%)
23	Osaka Habikino Medical Center	Osaka	306 (23.3%)
24	Shimizu Family Clinic	Osaka	21 (1.6%)
		Total	1315

Data are presented as the number and percentage of the parents.
